# Gastric cancer mortality trends in Spain, 1976-2005, differences by autonomous region and sex

**DOI:** 10.1186/1471-2407-9-346

**Published:** 2009-09-28

**Authors:** Esther García-Esquinas, Beatriz Pérez-Gómez, Marina Pollán, Elena Boldo, Pablo Fernández-Navarro, Virginia Lope, Enrique Vidal, Gonzalo López-Abente, Nuria Aragonés

**Affiliations:** 1 Department of Preventive Medicine. Ramón y Cajal Hospital. Madrid. Spain; 2 Environmental and Cancer Epidemiology Unit. National Center for Epidemiology, Carlos III Institute of Health. Madrid, Spain; 3 Consortium for Biomedical Research in Epidemiology & Public Health (CIBER en Epidemiología y Salud Pública - CIBERESP), Spain

## Abstract

**Background:**

Gastric cancer is the second leading cause of oncologic death worldwide. One of the most noteworthy characteristics of this tumor's epidemiology is the marked decline reported in its incidence and mortality in almost every part of the globe in recent decades. This study sought to describe gastric cancer mortality time trends in Spain's regions for both sexes.

**Methods:**

Mortality data for the period 1976 through 2005 were obtained from the Spanish National Statistics Institute. Cases were identified using the International Classification of Diseases 9^th ^and 10^th ^revision (codes 151 and C16, respectively). Crude and standardized mortality rates were calculated by geographic area, sex, and five-year period. Joinpoint regression analyses were performed to ascertain whether changes in gastric cancer mortality trends had occurred, and to estimate the annual percent change by sex and geographic area.

**Results:**

Gastric cancer mortality decreased across the study period, with the downward trend being most pronounced in women and in certain regions situated in the interior and north of mainland Spain. Across the study period, there was an overall decrease of 2.90% per annum among men and 3.65% per annum among women. Generally, regions in which the rate of decline was sharpest were those that had initially registered the highest rates. However, the rate of decline was not constant throughout the study period: joinpoint analysis detected a shift in trend for both sexes in the early 1980s.

**Conclusion:**

Gastric cancer mortality displayed in both sexes a downward trend during the study period, both nationally and regionally. The different trend in rates in the respective geographic areas translated as greater regional homogeneity in gastric cancer mortality by the end of the study period. In contrast, rates in women fell more than did those in men. The increasing differences between the sexes could indicate that some risk factors may be modifying the sex-specific pattern of this tumor.

## Background

Gastric cancer (GC) is the second leading cause of oncologic death worldwide [1,2], due to its elevated incidence and lethality; in many countries survival at five years of diagnosis is under 20% [3]. This cancer, with higher rates in males and sex ratios generally close to 2, displays wide geographic variability. In Japan, China and some regions of Eastern Europe and Latin America (Chile, Ecuador), rates are almost 20 times higher than those registered in India, the Philippines, USA or some areas in North and East Africa [1,2].

One of the most noteworthy characteristics of this tumor's epidemiology is the marked decrease seen in its incidence and mortality since the second half of the 20^th ^century: whereas stomach cancer was the second leading cause of death due to malignant tumors in the European Community in the 1990s [4], it currently ranks fourth after tumors of lung, breast and colon [2]. In Europe, gastric cancer incidence and mortality have registered a downward trend since the 1960s [5,6].

Generally, this downward turn in gastric cancer is attributed to changes in some risk factors traditionally implicated in its etiology, namely: improvements in the way in which food is preserved, including the use of refrigeration, changes in the dietary habits of the population, and the decline in the prevalence of *Helicobacter pylori *infection [7-9]. Yet, as is the case with most tumors, the etiology of gastric cancer has not been entirely elucidated, in that there are other risk factors referred to in the literature, such as socio-economic level [10], presence of contaminants in drinking water [11,12], and certain occupational exposures [13,14].

In Spain, the trend in GC mortality rates in recent years has been similar to that of other countries in Western Europe. This tumor is currently the 5^th ^leading cause of cancer-related death in both sexes, with rates of 12.94 and 5.65 in men and women, respectively [15]. However, as has also been reported in other European countries, such as Greece and Italy [16], there are important geographic differences within Spain. In this sense, some Spanish regions present mortality rates that are much higher than the European mean, while others show rates that are among the lowest in the continent [17,18]. This study sought to describe GC mortality time trends in Spain and its constituent regions, for the period 1976 through 2005.

## Methods

Mortality data were drawn from individual death entries furnished by the National Statistics Institute (NSI). We included all deaths certified as stomach cancer (codes 151 and C16 of the International Classifications of Diseases, 9^th ^and 10^th ^revisions (ICD-9 and ICD-10), respectively) in the period 1976-2005. As denominators, we used populations estimated at July 1 of each year, also provided by the NSI.

Crude and standardized mortality rates were calculated by the direct method (standard European population) for each Autonomous Region (AR), sex and five-year period (1976-1980,...; 2000-2005).

Trends in mortality rates (national and regional) were calculated by estimating the annual percent change (APC) and studying possible joinpoints (points at which a change in trend during the period is observed) in the trend. For this purpose, crude and standardized rates were then calculated for each calendar year, again broken down by AR, sex, and broadly categorized age group (35-64 and over 64 years).

Joinpoint analysis is known as multiple-phase regression analysis and is based on a Poisson model that fits the observed mortality rates [19]. Starting with the model having the minimum number of joinpoints the program adds new joinpoints (up to a maximum of three), and ascertains whether these should or should not be included in the initial model on the basis of their significance. To this end it performs hypothesis tests among the different models, based on a Monte Carlo method [20,21]. The final model estimates the joinpoints and the APC for each temporal segment displaying constant trend. Each joinpoint indicates a significant change in trend during the study period.

## Results

Table 1 shows the standardized five-yearly mortality rates for Spain as a whole and for each of the ARs, grouped by trend pattern registered over the study period and geographic location. Higher mortality rates were registered at the beginning of the period, with these being particularly high in some regions in inland and Northern Spain, with Castile & León at the head. Across the period analyzed, mortality decreased in all regions of Spain. This decline was more pronounced in those areas which initially had the highest rates, something that translated as greater regional homogeneity in GC mortality by the end of the study period. Hence, in the first five-year period, the observed rates ratio among the regions with highest and lowest mortality (Castile & León and the Balearic Islands, respectively) was 2.53 for men and 3.54 for women, while in the last five-year period these ratios were 1.99 and 2.10, respectively. Despite the declining trends, central and Northern Spain continued to be the regions with the highest rates in 2005, with Castile & León again at the head. In terms of gender, mortality was always higher in males, both nationally and regionally.

**Table 1:** Gastric cancer mortality in Spain (1976-2005): age-adjusted mortality rates per 100,000 person-years (European standard population).

		1976-1980	1981-1985	1986-1990	1991-1995	1996-2000	2001-2005
***MEN***							
**SPAIN**		**32.12**	**25.7**	**23.00**	**20.00**	**17.42**	**15.14**
Inland ARs^1^	Castile & León	46.98	36.53	31.81	28.40	24.31	20.14
	Rioja	34.52	31.68	24.38	21.52	20.28	14.67
	Aragon	31.94	26.77	23.60	18.53	15.45	14.10
	Castile-La Mancha	37.20	28.69	25.96	20.91	18.11	15.95
	Extremadura	40.39	31.01	26.31	21.14	18.72	17.89
	Madrid	26.01	19.84	19.50	18.44	16.27	14.07
Northern ARs^1^	Galicia	37.68	30.11	28.38	24.02	21.22	17.39
	Navarre	39.76	29.57	27.01	23.53	18.66	16.13
	Asturias	33.50	27.45	23.84	18.97	19.06	15.72
	Cantabria	30.77	22.37	21.25	18.26	17.74	14.36
	Basque Country	37.03	30.60	27.05	24.85	21.10	17.83
Eastern ARs^1 ^(Levante)	Valencia	27.71	22.53	22.25	18.76	17.26	14.50
	Catalonia	28.41	23.18	21.01	18.39	15.36	14.31
	Murcia	26.16	22.98	18.26	15.14	15.48	13.44
Southern ARs^1^	Andalusia	29.47	23.96	19.75	16.98	15.16	13.87
Islands	Balearic Islands	18.58	15.03	13.44	14.57	13.44	10.10
	Canary Islands	21.64	18.97	15.46	14.15	12.41	10.38
Ceuta/Melilla	Ceuta	34.26	34.62	23.32	14.63	16.12	14.9
	Melilla	27.67	21.07	14.51	10.97	8.10	11.50

***WOMEN***							
**SPAIN**		**16.33**	**12.76**	**11.07**	**9.13**	**7.50**	**6.43**
Inland ARs^1^	Castile & León	26.95	19.82	16.18	13.26	10.13	8.85
	Rioja	18.80	13.71	11.61	11.95	6.46	8.22
	Aragon	18.57	14.23	11.60	9.82	7.81	6.37
	Castile-La Mancha	19.98	16.01	12.12	9.76	8.04	6.76
	Extremadura	18.66	15.79	12.22	9.85	8.04	5.85
	Madrid	12.80	9.94	10.17	8.61	7.15	6.11
Northern ARs^1^	Galicia	20.08	16.52	14.58	11.63	9.94	8.33
	Navarre	19.77	13.00	13.36	9.32	7.82	6.90
	Asturias	18.18	13.84	12.44	9.68	7.89	6.54
	Cantabria	14.93	11.02	10.09	7.18	6.46	6.19
	Basque Country	17.20	13.19	11.90	9.51	7.74	6.64
Eastern ARs^1 ^(*Levante*)	Valencia	14.51	11.57	10.56	8.68	7.15	6.54
	Catalonia	14.37	11.37	9.94	8.43	6.85	5.84
	Murcia	14.17	10.11	8.87	8.76	7.28	6.46
Southern ARs^1^	Andalusia	13.25	10.75	8.86	7.04	6.39	5.46
Islands	Balearic Islands	7.62	7.48	7.47	7.00	4.65	4.21
	Canary Islands	11.80	8.12	6.33	6.45	5.51	4.53
Ceuta/Melilla	Ceuta	17.17	11.38	11.71	12.42	6.17	4.60
	Melilla	15.81	10.89	10.35	11.93	6.05	6.30

^1 ^ARs: Autonomous Regions.

**Figure 1:**
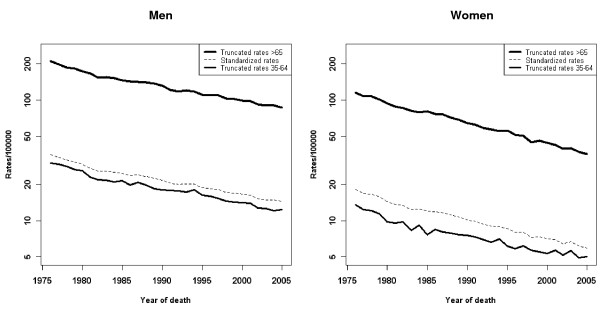
**Trend by sex in standardized and truncated rates and cases observed across the period**.

Figure 1 depicts the trend of standardized and truncated rates (ages 35-64 years and 65 years or more). Note should be taken of the steeper downward trend among women aged over 65 years. [see Additional file 1] shows the APC for the entire period and for the last 10 years, together with the rates for the first and last quinquennia. Also shown are the inflection points detected and the trend for each time segment. Across the study period, there was an overall decrease in the rates of 2.90% per annum in men and 3.65% per annum in women. Joinpoint analysis detected a change in trend in both sexes in the early 1980s. During the first period, rates fell by 4.58% per annum in men and 5.24% per annum in women, with the rate of decline leveling off thereafter.

**Figure 2:**
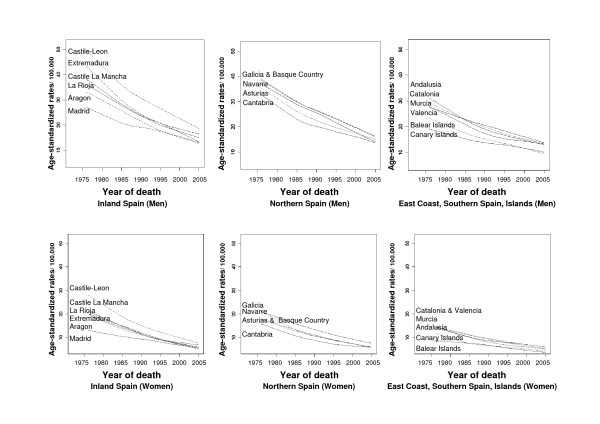
**Trend in gastric cancer mortality from 1975 to 2005, by area and sex**.

**Figure 3:**
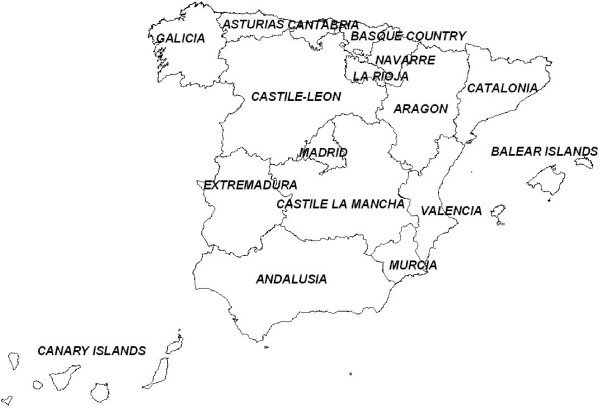
**Spanish Autonomous Communities**.

When the trend was analyzed in the individual ARs (excluding the autonomous city enclaves of Ceuta and Melilla), two mortality patterns were discerned (Figure 2). The first of these included the ARs situated in the interior and in Northern Spain (Castile & León, Castile-La Mancha, Extremadura, La Rioja, and Aragon), with the highest rates at the beginning of the period and the greatest overall rate of decline during the initial years studied (APC of 2.7% to 3.6% in males, and 3.4% to 4.4% in women). The second group included the ARs in Eastern Spain, Andalusia and the Canary and Balearic Islands, with the lowest rates and, in general, lower rates of decline. The Madrid Region registered a pattern unlike that of the other ARs in the center of the country, with its trend being more like that of Eastern Spain. As reference, figure 3 shows the situation of Spain's Autonomous Communities.

Finally, it should be noted that the decline in the rates was greater among females, a phenomenon observed throughout the period. This larger rate of decline in women (who started from lower rates) explains the fact that sex-related differences have become more pronounced in recent years. Indeed, in some areas this has been compounded by the slowdown observed in the decrease in male mortality during the last ten years (e.g. Extremadura, Andalusia and Catalonia).

## Discussion

Our results highlight a decrease in GC mortality in Spain across the entire study period. This fall proved greatest in areas which initially had the highest rates (inland areas lying near the Cantabrian mountains and the central plateau), a phenomenon that has made for greater uniformity among the different regional rates over time. Castile & León maintain the highest rates, with an especially high degree of excess mortality in men. Despite having started from lower rates, women nonetheless registered a greater decline in mortality rates due to this tumor.

When it comes to interpreting these results, the limitations of mortality data must be borne in mind. To study cause-specific trends, the best approach would be to combine incidence and mortality data. As there is no information on GC incidence countrywide, mortality continues to be the sole comprehensive source of information in Spain. In the case of GC, the fact that relative survival at 5 years is low, i.e., 24.9% in Europe and 31.8% in Spain [22], means that mortality data are a relatively reliable approximation of incidence rates. However, some authors suggest that in some countries the decline in mortality might have been swifter than that of incidence, in some countries at least [23]. Available incidence data from regional cancer population registries also suggest that this might be the case in Spain [24].

A further possible limitation of the data used is linked to the quality of death registry entries. In Spain, a recent review indicated that, for GC, mortality statistics are a valid and useful tool for estimating this tumor's impact [25]. Nevertheless, there are discontinuities in the coding which could be responsible for some of the changes observed in trends. The change in coding from ICD-8 to ICD-9 was introduced around 1980, and could be related with the change in trend observed for the country in the early 80s. In addition, the decentralization in the coding of mortality statistics in this decade led to an increase in the quality of these data [25], which might be responsible for some of the shifts in trends, as could be the case of Madrid. The improvement in the coding of some gynecological neoplasms (of ovarian or uterine origin), which have sometimes been erroneously registered as tumors of other organs of the abdominal cavity such as stomach, could have had some degree of influence on the sharper decline in trends among women [25].

To delimit the true causes that have led to the observed differences in GC trends among ARs is complex. Some factors, such as the role of *H. pylori *as a carcinogenic promoter [26], or diet [27,28], which contribute to the etiopathogeny of GC are widely accepted. In Spain, studies published on the prevalence of *H. pylori *infection report interregional differences ranging from 43% [29] to 69% [30], but the scarcity of data do not enable their implication in the trend in rates to be assessed. Insofar as diet is concerned, some risk factors, e.g., intake of nitrates through consumption of cured and pickled foods [31,32] or drinking water [33,34], and prevention factors, e.g., consumption of fruit and vegetables in particular, have been recognized. Along with these factors, others have been described, such as socio-economic differences, a variable that may in turn indicate differences in exposure to various factors including prevalence of *H. pylori *infection, diet, tobacco use, alcohol, or access to the health system, among others [23].

During the study period, Spain's society underwent major transformations. Vigorous economic development was accompanied by important changes in social structure, with the urban middle classes becoming predominant, and increases in *per capita *income and the percentage allocated to public health expenditure [35,36]. All these changes, taken together with a notable improvement in lifestyles, took place in parallel with the trend plotted by gastric cancer mortality.

In terms of socio-economic level, uniformity in rates has been accompanied by a convergence in socioeconomic levels among the different ARs [37,38]. Even so, if one examines the economic level of each AR, it will be noted that among the ARs which had a lower socioeconomic level in the mid-20^th ^century, some are still registering the highest GC mortality rates 25-30 years afterwards (Extremadura, Castile & León, Castile-La Mancha, and Galicia), whereas in others (Murcia, Andalusia) the opposite has occurred. Similarly, areas in which GDP *per capita *was higher than the national mean in the 1970s, such as Navarre or La Rioja, register elevated mortality rates.

In general terms, one of the reasons that may account for the decrease in mortality rates is the change in diet, with a progressive globalization in food consumption patterns and greater accessibility to perishable foods [39]. Whereas Spaniards' food intake was very unequal at the beginning of the century, being characterized by protein, mineral and vitamin deficiency [40], the nutritional transition, initiated with the industrialization process, entailed a change in the consumption of basic foods and an increase in foods of animal origin, fruit, and sugars. Homemade food production thus fell and industrial production rose. An important element in this change was the introduction of the first refrigerators in Spain in the early 1950s, though these were to take some years in finding their way into all Spanish homes. Little by little, habits tend to become more uniform, and differences in terms of consumption of certain foods, such as fruit and vegetables, viewed as protective against GC by providing compounds with antioxidant properties, tend to disappear [39,41,42].

With respect to *H. pylori*'s role as a carcinogenic promoter, it has not been possible to measure the impact of *H. pylori *eradication treatment on the decrease in GC incidence [43], yet there is strong evidence to show that administration of such treatment in certain subgroups of patients prevents the progression of precursor lesions [44]. Sight should therefore not be lost of the favorable effect that the introduction and progressive improvement in *H. pylori *treatment guidelines, coupled with the generalized use of antibiotics which took place in the latter half of the 20^th ^century, may have had on the observed trends.

Along these same lines, the role of probiotics in the prevention and eradication of *H. pylori *has recently been proposed [45-49]. The population's growing concern with health has made the so-called "functional foods" fashionable (i.e., foods enriched with biologically activated components); probiotics constituting up to 65% of the European functional food market [50]. Specifically, in Eastern Spain a reduction in the consumption of liquid milk in favor of enriched dairy products has been observed [51,52], fundamentally among the higher-level social classes [53]. This also occurs in the Canary Islands [54] and Andalusia, where these products have traditionally been less expensive and the habit of consuming such foods is better established. In contrast, in the North and North-west regions and in Castile-La Mancha, the consumption of liquid milk still takes precedence.

Another factor that could be implicated in the decline in mortality is the improvement in drinking-water quality, linked to the country's economic development. Thus, in the study period, the population supplied by surface waters increased, in detriment to the use of subterranean waters which have a lower power of recovery vis-à-vis the presence of contaminants [55]. Besides, quality criteria set by international bodies became more stringent [56], thereby contributing to the decrease in exposure to physical or biological chemical contaminants in the Spanish population [57].

Lastly, stress must again be laid on the differences between the two sexes observed in the trend. To explain some of the sex-related differences in GC distribution, the protective role of estrogenic hormones has been proposed. These differences would not, however, account for the different behavior observed in the rates of decline, bearing in mind that when these rates were truncated, these differences were seen to become even more pronounced in women aged over 65 years. The role that improvements in coding may have had on this finding, has already been discussed above. Nevertheless, in view of the dominant role of environmental factors in the appearance of GC, consideration should be given to the possible coexistence of other factors of unequal sex-related distribution which might be influencing the two groups differently. Some authors propose greater self-care among women, or cite the different effect that physical exercise may have in both sexes, e.g., a recently published paper proposes the beneficial role of an increase in physical activity on reduction in GC risk among women, with no such effect being found for gastric cancer among males [58].

## Conclusion

In conclusion, gastric cancer displayed a downward trend throughout the study period, both for Spain overall and for all the country's constituent regions, across the sexes. The different trend registered in the Spanish ARs has, however, translated as greater regional homogeneity in mortality due to this tumor because the fall was steeper in areas which initially had the highest rates. It is essential that efforts continue to be directed at ascertaining which factors are determining the greater rate of decline among women. Their identification could amount to an important advance in the prevention of this tumor.

## Abbreviations

GC: Gastric cancer; APC: Annual percent change; AR: Autonomous region.

## Competing interests

The authors declare that they have no competing interests.

## Authors' contributions

NA, BPG, MP and GLA were all involved in designing the study. EGE, NA and BPG performed the statistical analysis and wrote the first draft of the manuscript to which all authors subsequently contributed. All authors read and approved the final manuscript.

## Pre-publication history

The pre-publication history for this paper can be accessed here:

http://www.biomedcentral.com/1471-2407/9/346/prepub

## Supplementary Material

Additional file 1 **Table S1**. Age-standardized cancer mortality rates per 100,000 person-years (1976-1980 and 2001-2005) and joinpoint analysis (1976-2005) in Spain, by sex and Autonomous Region. Click here for file
