# Supplementary material for: Gastric cancer mortality trends in Spain, 1976-2005, differences by autonomous region and sex
Source: BMC Cancer. 2009 Sep 28;9:346. doi: 10.1186/1471-2407-9-346 (PMC2761406; doi:10.1186/1471-2407-9-346)
Supplement: Additional file 1 — Table S1. Age-standardized cancer mortality rates per 100,000 person-years (1976-1980 and 2001-2005) and joinpoint analysis (1976-2005) in Spain, by sex and Autonomous Region. [file 1471-2407-9-346-S1.DOC]

**Additional files:**

Additional file 1

File format: DOC
Title: Age-standardized gastric cancer mortality rates and joinpoint analysis in Spain (1976–2005)

Description: Age-standardized cancer mortality rates per 100,000 person-years (1976–1980 and 2001–2005) and joinpoint analysis (1976–2005) in Spain, by sex and Autonomous Region.

|  | | Age-standardized  mortality rates | | APC | APC | Joinpoint analysis (1976-2005) | | | | | | |
| --- | --- | --- | --- | --- | --- | --- | --- | --- | --- | --- | --- | --- |
|  | | 1976-1980 | 2001-2005 | 1976-2005 | 1995-2005 | Joinpoints | Trend 1 | | Trend 2 | | Trend 3 | |
|  |  |  |  |  | (last 10 years) |  | Years | APC | Years | APC | Years | APC |
| ***MEN*** |  |  |  |  |  |  |  |  |  |  |  |  |
| **SPAIN** |  | **32.12** | **15.14** | **-2.90*** | **-2.6*** | **1** | **76-82** | **-4.58*** | **83-05** | **-2.61*** |  |  |
| Interior ARs | Castile & León | 46.98 | 20.14 | -3.37* | -3.15* | 0 |  |  |  |  |  |  |
|  | Rioja | 34.52 | 14.67 | -3.29* | -3.29* | 0 |  |  |  |  |  |  |
|  | Aragon | 31.94 | 14.10 | -3.35* | -3.35* | 0 |  |  |  |  |  |  |
|  | Castile-La Mancha | 37.20 | 15.95 | -3.35* | -3.35* | 0 |  |  |  |  |  |  |
|  | Extremadura | 40.39 | 17.89 | -3.36* | -1.8* | 1 | 76-92 | -4.30* | 92-05 | -1.84* |  |  |
|  | Madrid | 26.01 | 14.07 | -2.13* | -2.5* | 2 | 76-85 | -5.70* | 85-88 | +6.32 | 88-05 | -2.51* |
| Northern ARs | Galicia | 37.68 | 17.39 | -2.85* | -2.85* | 0 |  |  |  |  |  |  |
|  | Navarre | 39.76 | 16.13 | -3.59* | -3.59* | 0 |  |  |  |  |  |  |
|  | Asturias | 33.50 | 15.72 | -2.87* | -2.87* | 0 |  |  |  |  |  |  |
|  | Cantabria | 30.77 | 14.36 | -2.72* | -2.72* | 0 |  |  |  |  |  |  |
|  | Basque Country | 37.03 | 17.83 | -2.75* | -2.75* | 0 |  |  |  |  |  |  |
| Eastern ARs (*Levante*) | Valencia | 27.71 | 14.50 | -2.39* | -2.39* | 0 |  |  |  |  |  |  |
|  | Catalonia | 28.41 | 14.31 | -2.69* | -1.10* | 2 | 76-02 | -2.88* | 02-05 | -2.63 |  |  |
|  | Murcia | 26.16 | 13.44 | -2.61* | -2.61* | 0 |  |  |  |  |  |  |
| Southern ARs | Andalusia | 29.47 | 13.87 | -3.01* | -1.9* | 1 | 76-92 | -3.73* | 92-05 | -1.94* |  |  |
| Islands | Canary Islands | 21.64 | 10.38 | -2.88* | -2.88* | 0 |  |  |  |  |  |  |
|  | Balearic Islands | 18.58 | 10.1 | -1.81* | -1.81* | 0 |  |  |  |  |  |  |
| ***WOMEN*** |  |  |  |  |  |  |  |  |  |  |  |  |
| **SPAIN** |  | **16.33** | **6.43** | **-3.65*** | **-3.5*** | **1** | **76-81** | **-5.24*** | **81-05** | **-3.46*** |  |  |
| Interior ARs | Castile & León | 26.95 | 8.85 | -4.44* | -4.10* | 1 | 76-81 | -7.26* | 81-05 | -4.06* |  |  |
|  | Rioja | 18.80 | 8.22 | -3.54* | -3.54 | 0 |  |  |  |  |  |  |
|  | Aragon | 18.57 | 6.37 | -4.09* | -4.09* | 0 |  |  |  |  |  |  |
|  | Castile-La Mancha | 19.98 | 6.76 | -4.38* | -4.38 | 0 |  |  |  |  |  |  |
|  | Extremadura | 18.66 | 5.85 | -4.39* | -4.39 | 0 |  |  |  |  |  |  |
|  | Madrid | 12.80 | 6.11 | -2.78* | -3.50* | 2 | 76-84 | -5.92* | 84-87 | +6.08 | 87-05 | -3.52* |
| Northern ARs | Galicia | 20.08 | 8.33 | -3.42* | -3.42 | 0 |  |  |  |  |  |  |
|  | Navarre | 19.77 | 6.90 | -4.13* | -4.13* | 0 |  |  |  |  |  |  |
|  | Asturias | 18.18 | 6.54 | -3.94* | -3.94* | 0 |  |  |  |  |  |  |
|  | Cantabria | 14.93 | 6.19 | -3.62* | -3.62* | 0 |  |  |  |  |  |  |
|  | Basque Country | 17.20 | 6.64 | -3.74** | -3.74* | 0 |  |  |  |  |  |  |
| Eastern ARs (*Levante*) | Valencia | 14.51 | 6.54 | -3.17* | -3.17* | 0 |  |  |  |  |  |  |
|  | Catalonia | 14.37 | 5.84 | -3.50* | -3.50 | 0 |  |  |  |  |  |  |
|  | Murcia | 14.17 | 6.46 | -2.87* | -2.87 | 0 |  |  |  |  |  |  |
| Southern ARs | Andalusia | 13.25 | 5.46 | -3.57* | -3.57* | 0 |  |  |  |  |  |  |
| Islands | Balearic Islands | 7.62 | 4.21 | -2.59* | -2.59* | 0 |  |  |  |  |  |  |
|  | Canary Islands | 11.80 | 4.53 | -3.43* | -2.50* | 1 | 76-80 | -13.91* | 80-05 | -2.52* |  |  |

* p<0.05 ; ARs= Autonomous Regions; APC= Annual percent change;
